# An online web-based assessment tool to monitor graduate medical trainee professionalism and supervision

**DOI:** 10.5116/ijme.5b1e.21ca

**Published:** 2018-06-22

**Authors:** Manuel C. Vallejo, Ahmed F. Attaallah, Linda S. Nield, Rebecca M. Elmo, Scott Cottrell, Norman D. Ferrari

**Affiliations:** 1 Department of Anesthesiology and Medical Education, West Virginia University School of Medicine, USA; 2 Department of Anesthesiology, Florida Hospital, Tampa, USA; 3 Department of Medical Education, West Virginia University School of Medicine, USA

**Keywords:** Web-based assessment, monitor, graduate medical trainee, professionalism, supervision

## To the Editor

Medical education is tasked to ensure a humanistic training environment where trainees are taught to manifest professionalism and effacement of self-interest to meet the needs of their patients.^1^^-^^3^ Graduate and undergraduate medical education communities are searching for tools to assess professionalism, supervision, and mistreatment. The creation of timely and readily available assessment tools to monitor these events and trigger interventions is of much interest to medical educators. We describe our use of an institutional online reporting tool or “button” to document and track professionalism, supervision and mistreatment across multiple specialty training programs with the purpose of determining the underlying reasons for tool activations, and to enhance our understanding of how this assessment tool can improve graduate medical trainee professionalism and mistreatment.

### Program Description

As a way to collect observations and monitor graduate medical trainee and faculty conduct at our academic medical centre, we created web-based online “button” forms that are available to graduate medical trainees, faculty and other healthcare professionals to disseminate observations about professionalism and quality of supervision to our graduate medical education office.^4^^,^^5^

Before online “button” inception, the graduate medical education office disseminated information about the anticipated launch of the new online website tools to all graduate medical trainees, faculty, hospital nursing leadership, staff, and Graduate Medical Education Committee members. They received detailed information regarding how to correctly complete these forms several months before the go-live date in July 2013 and reminded them afterwards at regular graduate medical trainee, faculty and staff meetings to ensure their engagement and that institutional-wide participation would happen in these assessment strategies.

The promotion for launching the new professionalism and mistreatment tools was advertised as "Press the Button", as the person accessing these forms would press a button-shaped desktop icon that opens the graduate medical education forms' website on any institutional-computer to start reporting an event. This website is also accessible from other computers and connected mobile devices where the "Button" shortcut can be added to personal devices. Any hospital graduate medical trainee, faculty, or staff person in the institution (all had online graduate medical education website access) who witnessed or personally experienced a reportable event was encouraged to activate the online forms by describing the incident in as much detail as possible. These electronic forms can be reported anonymously or by self-identification. Graduate medical education leadership immediately reviewed a completed form, then forwarded to the programme director (for graduate medical trainee), to the department chair (for faculty activations), or to the director of medical staff affairs (for the non-graduate medical trainee, non-departmental activations). A written summary of actions and remediation is required to be sent back to graduate medical education within ten business days. The event remains open until a written summary of actions is received by the graduate medical education office and subsequently closed.

As a way to perform quality improvement and assurance on the button activations, we conducted an observational analysis of these activations from July 2013 (button inception) through July 2017.  Over this four-year-period there were 315 button activations, most were anonymous (69.0%) with graduate medical trainees being the primary offender (74%) in their first and second year of graduate medical education (21.9%; 21.6%). Having this readily available and easily accessible web-based tool allowed for a high rate of reporting and participation, and early intervention of unprofessional behavior. We believe continuing this process will encourage professionalism within the institution and subsequently lead to decreased incidents in an improved professional training environment.

Competence to practice medicine includes the ability of physicians to demonstrate professionalism in all relationships, including honesty to patients, patient confidentiality, maintaining appropriate patient relations, improving the quality of patient care and access to care, having the ability to work with finite resources, with applicable scientific knowledge and principals.^2^^,^^6^^,^^7^ Demonstrating professionalism and ensuring a humanistic learning environment for medical trainees is important at all levels.^3^^,^^6^ Identifying and correcting deficits in professionalism early in residency benefits the trainee, the patient and humanity.^2^^,^^6^ Any system for assessing professionalism should be transparent, anonymous, behavior-focused, low cost, and allow for formative feedback with focused intervention and remediation.^6^^,^^8^  This online web-based assessment tool encompasses all these features and can be readily used to monitor professionalism and supervision-mistreatment.

### Conflicts of Interest

The authors declare that they have no conflict of interest.
